# Colorectal cancer screening in individuals with multiple sclerosis: A mixed-methods study

**DOI:** 10.1177/13524585261465197

**Published:** 2026-08-07

**Authors:** Chloé Pierret, Romane Bouvier, Sandrine Kerbrat, Emmanuelle Leray

**Affiliations:** Rennes University, École des Hautes Etudes en Sante Publique, Rennes, France; Rennes University, École des Hautes Etudes en Sante Publique, Rennes, France; DAMAD, Plouzané, France; Rennes University, École des Hautes Etudes en Sante Publique, Rennes, France

**Keywords:** Multiple sclerosis, cancer screening, mixed methods, colorectal cancer, medico-administrative databases

## Abstract

**Introduction:**

People with multiple sclerosis (pwMS) have a lower reported risk of colorectal cancer, which may reflect reduced access to colorectal cancer screening (CCS).

**Objectives:**

Compare CCS in pwMS and the general population and identify barriers and facilitators using mixed methods.

**Methods:**

In 2022–2024, PwMS aged 50–74 years and matched controls (3:1) were selected. Overall and age-stratified CCS coverage rates were computed. In pwMS, multivariable mixed-effects logistic models identified factors associated with CCS ⩾ 80%. Semi-structured interviews were carried out with 20 pwMS.

**Results:**

The cohort included 63,509 pwMS and 190,527 controls (72.0% women, mean age 59.2 years). Annual CCS coverage was 41.2% in pwMS and 42.4% in controls (*p* < 0.001). In pwMS, CCS was higher in the 50–54 years (+1.5%, *p* < 0.001) and lower in the ⩾65 years group (−1.6% to −6.2%, *p* < 0.001). Male sex (odds ratio = 0.90; 95% CI = [0.86–0.93]) and lower ecological socioeconomic status (0.81 [0.76–0.86]) were associated with lower CCS, while disease-modifying therapies (1.12 [1.08–1.16]) and high healthcare use with higher CCS. Qualitative barriers included motor or digestive MS symptoms and feelings of disgust. Facilitators included health system trust and prevention awareness.

**Conclusion:**

CCS is lower among older pwMS and remains suboptimal in both pwMS and controls.

## Introduction

Colorectal cancer (CRC) is among the most common cancer types and the second leading cause of cancer-related mortality in the general population. In people with multiple sclerosis (pwMS), it remains an important cause of morbidity and mortality.^1,2^ Research on CRC in pwMS reported inconsistent results: although incidence studies show either a reduction^1,3^ or no difference in CRC risk,^4–6^ mortality studies report increased CRC-related mortality among pwMS.^7,8^ CRC survival strongly depends on early detection and timely treatment, and lower access to colorectal cancer screening (CCS) in pwMS may lead to a higher proportion of delayed-diagnosis or symptom-detected cancer, explaining lower incidence but higher mortality.^9^ A meta-analysis on cancer inequalities showed higher mortality rates and consistently lower screening uptake for breast, CRC, and cervical cancer in individuals with mental and physical disability,^10^ including multiple sclerosis (MS), which is the leading cause of disability in young adults. However, a Canadian case–control study observed no difference between pwMS and matched controls in CRC diagnosis route (screening vs symptomatic) and time to diagnosis. However, the small number of pwMS with CRC (*n* = 54) may have limited the ability to detect modest differences or draw generalizable conclusions.^11^ Our group recently showed lower breast cancer screening (BCS) rates in pwMS compared to the French general population, especially in older individuals.^12^ Perceived barriers included feelings of overmedicalization and accessibility issues (transportation, procedure, scheduling conflicts) that may also affect CCS uptake. Therefore, population-based studies are needed to quantify CCS access in pwMS and to identify the factors associated with lower uptake and the underlying mechanisms.

The objective of this study was to assess and compare CCS rates between pwMS and the general population and to identify barriers and facilitators of CCS uptake using a mixed-methods approach.

## Methods

### Study design

This study used a convergent mixed-methods design combining quantitative and qualitative analyses to examine the research question: “What are the barriers and facilitators to CCS in MS?.” A parallel analysis approach was used, with both components collected and analyzed separately, and then integrated at the interpretation stage.^13^ Reporting followed the Good Reporting of A Mixed Methods Study (GRAMMS) checklist (Supplemental Table S1).^14^

### Quantitative analysis

#### Population and design

A 2022–2024 population-based retrospective matched cohort study was performed using data from the Système National des Données de Santé (SNDS), the French national administrative healthcare database (~99% of the population).^15^ It collects exhaustive data on healthcare consumption in France, including hospitalizations (in- and out-patient), ambulatory care, and screening procedures. The aim was to evaluate and compare CCS coverage rates in pwMS and in a matched control group.

Individuals with prevalent MS on 31 December 2021 were identified in the SNDS database with a previously used algorithm^1,12,16,17^ based on the long-term disease (LTD) status, MS-related hospitalizations, and MS-specific drug prescriptions.^18^ Selection criteria were being eligible for CCS (50–74 years) and without a history of chronic inflammatory bowel disease or cancer (any type) in the past 3 years.

The follow-up start, defined as the index date, began on 1 January 2022 or 50th birthday. Each patient was exactly matched to three controls from the general population on birth year, health insurance status, residence area, and index date. All participants were followed until the first censoring event: 31 December 2024, 75th birthday, first cancer occurrence (not restricted to CRC), or death.

#### Main outcome: CCS coverage

The CCS coverage rate was defined as follows:

$$\frac{\mathrm{Number}\;\mathrm{of}\;\;\mathrm{days}\;\;\mathrm{during}\;\;\mathrm{which}\;\;\mathrm{an}\;\;\mathrm{individual}\;\;\mathrm{is}\;\;\mathrm{considered}\;\;\mathrm{covered}\;\;\mathrm{by}\;\;a\;\;\mathrm{CCS}\;\;\mathrm{procedure}}{\mathrm{Number}\;\;\mathrm{of}\;\;\mathrm{days}\;\;\mathrm{during}\;\;\mathrm{which}\;\;\mathrm{an}\;\;\mathrm{individual}\;\;\mathrm{is}\;\;\mathrm{eligible}\;\;\mathrm{for}\;\;\mathrm{CCS}}*100$$

Two medical procedures were considered: fecal immunochemical test (FIT), which is the recommended screening tool to be performed every 2 years, and colonoscopy, which is done every 5–10 years and is usually recommended for surveillance in people with personal or familial history of CRC or high-risk profiles (Supplemental Table S2). To accurately assess CCS coverage, colonoscopies from 2012 to 2024 and FIT tests from 2020 to 2024 were retrieved.

#### Factors associated with CCS

Variables potentially associated with CCS included socio-demographic, MS-related and overall health, healthcare utilization, and MS management indicators.

Socio-demographic factors at inclusion were age, sex, department of residence, FDep index (a city-level social deprivation index; quasi-log-linear relationship with mortality),^19^ and CMU/C2S status (social deprivation index at the individual level based on income). MS-related and overall health variables were computed in the 12 months preceding inclusion and comprised the Bannay-Charlson comorbidity index (CCI; from hospitalization, medical procedures, LTD-status and drug reimbursement, and excluding cancer and hemiplegia; Supplemental Table S3),^20^ hemiplegia identified through hospitalization data (derived from the CCI but entered separately), MS duration (time from SNDS identification to inclusion), and MS-related overnight hospitalizations. Healthcare utilization indicators included general practitioner (GP), nursing, physiotherapy, and pharmacy visits and ambulatory (i.e. 1-day) hospitalizations. Disease-modifying therapy (DMT) reimbursements in the previous year were retrieved as a proxy of MS management. All variables were computed at the index date.

#### Statistical analysis

CCS coverage rates and their 95% confidence intervals (CI) were calculated and compared between pwMS and controls, overall and stratified by age, sex, comorbidity status, and FDep quintile. Age- and sex-standardized CCS coverage rates were also stratified by MS duration. The proportion of individuals with no CCS coverage (0%), partial coverage (1%–79%), and adequate coverage (⩾80%) was compared according to age and MS duration.

Factors that could act as barriers and facilitators and were associated with CCS were evaluated only in pwMS with complete data. The main outcome of interest was having a CCS coverage ⩾80%.^21^ Associations were assessed using a multivariable mixed-effects logistic model with a random effect on the region of residence. A sensitivity analysis was conducted using different CCS coverage rate thresholds (50% and 0%).

### Qualitative analysis

Semi-structured interviews were conducted with pwMS eligible to the organized CCS. Recruitment was conducted through a call for testimonies on social media and MS association websites. To ensure a wide range of screening behaviors, the call only mentioned preventive services in the context of MS and not CCS. Among 31 eligible volunteers, 20 were selected to maximize the profile diversity (age, sex, MS duration, screening behaviors, employment status, region of residence). After obtaining verbal informed consent, each interview was recorded using Teams or a microphone and transcribed with the ShareDocs platform (one participant refused to be recorded, manual notes were used).^22^ After anonymization and transcription, the recordings were deleted.

An interview grid was developed to address, particularly, the following topics: healthcare pathways, general knowledge about CRC, its screening program and procedure, general knowledge of CCS prevention campaigns, perceived barriers and facilitators to CCS, and the perceived role of healthcare professionals in CCS (Supplemental Table S4). Barriers and facilitators were identified using an empirically inductive thematic method in which patterns or themes are identified and analyzed in qualitative data without imposing preconceived theories or categories.^23^ Key topics were identified first individually, then reviewed and combined (R.B. and C.P.). Interviews were conducted until thematic saturation was reached.

The quantitative and qualitative data were analyzed separately and integrated (C.P. and R.B.) through the identification and reporting of convergencies, divergencies, complementarities (different but not contradictory), and expansions (overlapping findings with space for additional interpretation).^24^

### Data access and ethics approval

Data access approvals were obtained according to the current French legislation (R.1461-11-R.1461-17, CNIL-2016-316) and the study was declared to the EHESP SNDS registry, equivalent to an Institutional Review Board approval.

## Results

### Quantitative results

#### Study population

**Figure 1.**
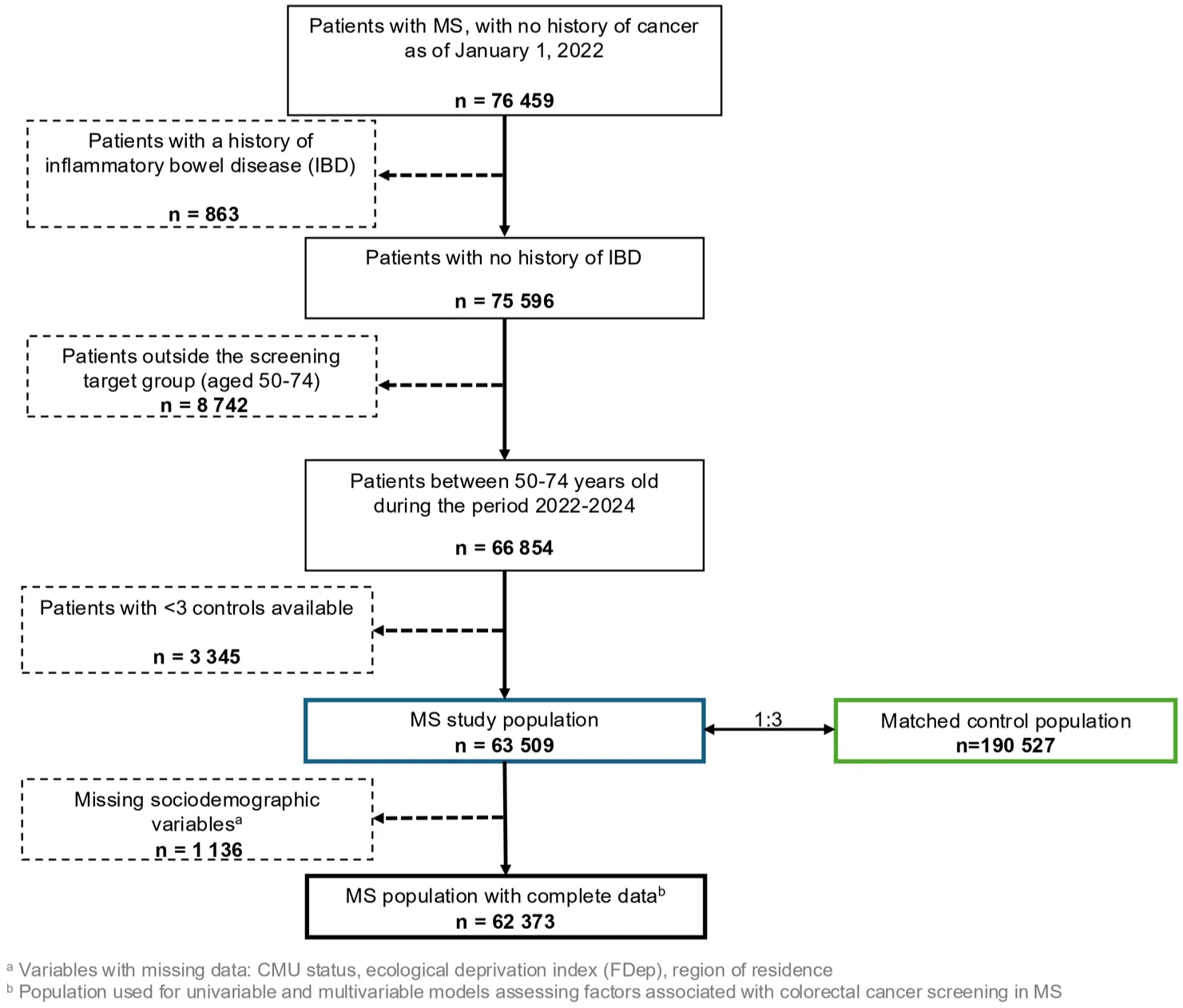
Study population flowchart. MS: multiple sclerosis; IBD: inflammatory bowel syndrome.

The cohort study included 63,509 pwMS matched to 190,527 controls (Figure 1). Their median age at the index date was 58 years (IQR, 53–65) and 72.0% were women. The median MS duration was 14.5 years (IQR, 8.6–21.5) and was ⩾10 years in 70.1% of participants (Table 1). In the year before the index date, 46.2% of pwMS received at least one DMT (high efficacy: 17.1%). Overall, 10.4% of participants had ⩾1 comorbidity.

**Table 1.** Study population characteristics at the index date (*n* = 254,036).

	pwMS	Controls	*p*
	*n* = 63,509	*n* = 190,527	
Socio-demographic characteristics	*N* (%)	*N* (%)	
Sex			N/A
Women	45,722 (72.0)	137,166 (72.0)	
Men	17,787 (28.0)	53,361 (28.0)	
Age, years			N/A
Median [IQR]	58 [53-65]	58 [53-65]	
50-54	20,447 (32.2)	61,341 (32.2)	
55-59	14,660 (23.1)	43,980 (23.1)	
60-64	12,446 (19.6)	37,338 (19.6)	
65-69	9239 (14.5)	27,717 (14.5)	
70-74	6717 (10.6)	20,151 (10.6)	
FDep (social deprivation index)			0.006
First quintile (least deprived)	12,196 (19.2)	35,793 (18.8)	
Second quintile	12,316 (19.4)	36,824 (19.3)	
Third quintile	12,875 (20.3)	37,896 (19.9)	
Fourth quintile	12,892 (20.3)	39,330 (20.6)	
Fifth quintile (most deprived)	13,111 (20.6)	40,119 (21.1)	
Missing	119 (0.2)	565 (0.3)	
CMU/C2S			<0.0001
Yes	4154 (6.5)	15,288 (8.0)	
No	58,518 (92.2)	171,460 (90.0)	
Missing	837 (1.3)	3779 (2.0)	
**MS-related characteristics**			
MS duration, years			
Median [IQR]	14.5 [8.6-21.5]		
0-4	8047 (12.7)		
5-9	10,965 (17.3)		
10-19	25,147 (39.6)		
⩾20	19,350 (30.5)		
Disease-modifying therapy*	29,331 (46.2)	1239 (0.6)	<0.0001
Moderate efficacy	19,081 (30.0)		
Glatiramer acetate	2741 (4.3)		
Teriflunomide	6450 (10.2)		
Azathioprine	658 (1.0)		
Cladribine	133 (0.2)		
Dimethyl fumarate	4466 (7.0)		
Beta-interferon	4176 (6.6)		
Methotrexate	634 (1.0)		
Mycophenolate mofetil	987 (1.6)		
High efficacy	10,888 (17.1)		
Fingolimod	4437 (7.0)		
Natalizumab	1167 (1.8)		
Ocrelizumab	3108 (4.9)		
Rituximab	1907 (3.0)		
Ofatumumab	369 (0.6)		
Ponesimod	134 (0.2)		
Number of treated patients, according to age (years)			
50-54	12,713 (43.3)		
55-59	7703 (26.3)		
60-64	5032 (17.2)		
65-69	2624 (8.9)		
70-74	1259 (4.3)		
MS-related hospitalization, overnight^a^	1655 (2.6)		
MS-related day hospitalization^a^	1839 (2.9)		
Hemiplegia	3855 (6.1)	833 (0.4)	<0.0001
Comorbidity*			
Charlson comorbidity index (excluding cancer and hemiplegia)			0.281
0	56,790 (89.4)	170,766 (89.6)	
1	5278 (8.3)	15,666 (8.2)	
2	1070 (1.7)	3047 (1.6)	
3+	371 (0.6)	1048 (0.6)	
Hospitalization, overnight^b^	7695 (12.1)	12,398 (6.5)	<0.0001
Hospitalization, ambulatory^b^	7986 (12.6)	15,704 (8.2)	<0.0001
Healthcare use*			
In-clinic healthcare practitioner visit, median [IQR]	6 [3-10]	4 [2-7]	<0.0001
Including general practitioner	4 [2-7]	3 [1-5]	<0.0001
Hospital healthcare practitioner visit, median [IQR]	1 [0-2]	0 [0-1]	<0.0001
Pharmacy visit	60,973 (96.0)	172,477 (90.5)	<0.0001
Use of at-home nursing services	44,492 (70.1)	103,993 (54.6)	<0.0001
Physiotherapy	30,384 (47.8)	40,959 (21.5)	<0.0001

CMU: universal medical coverage; IQR: interquartile range; MS: multiple sclerosis.

a Hospitalization with a primary or related diagnosis = G35 (ICD-10 code for multiple sclerosis).

b Hospitalization with a primary or related diagnosis ≠ G35.

* In the 12 months before the index date.

#### CCS coverage rates

Mean CCS coverage rate was 41.2% (95% CI = 40.9–41.6) in pwMS and 42.4% (42.2–42.6) in controls, corresponding to a 3% relative decrease in pwMS (*p* < 0.0001). A substantial proportion had no CCS coverage during the study period (46.2% vs 44.5% in pwMS and controls, respectively; *p* < 0.0001). Among individuals with any coverage (⩾1 day), mean coverage was similar between groups (76.7% vs 76.4%, *p* = 0.2), indicating that the overall difference was driven by individuals without coverage.

**Figure 2.**
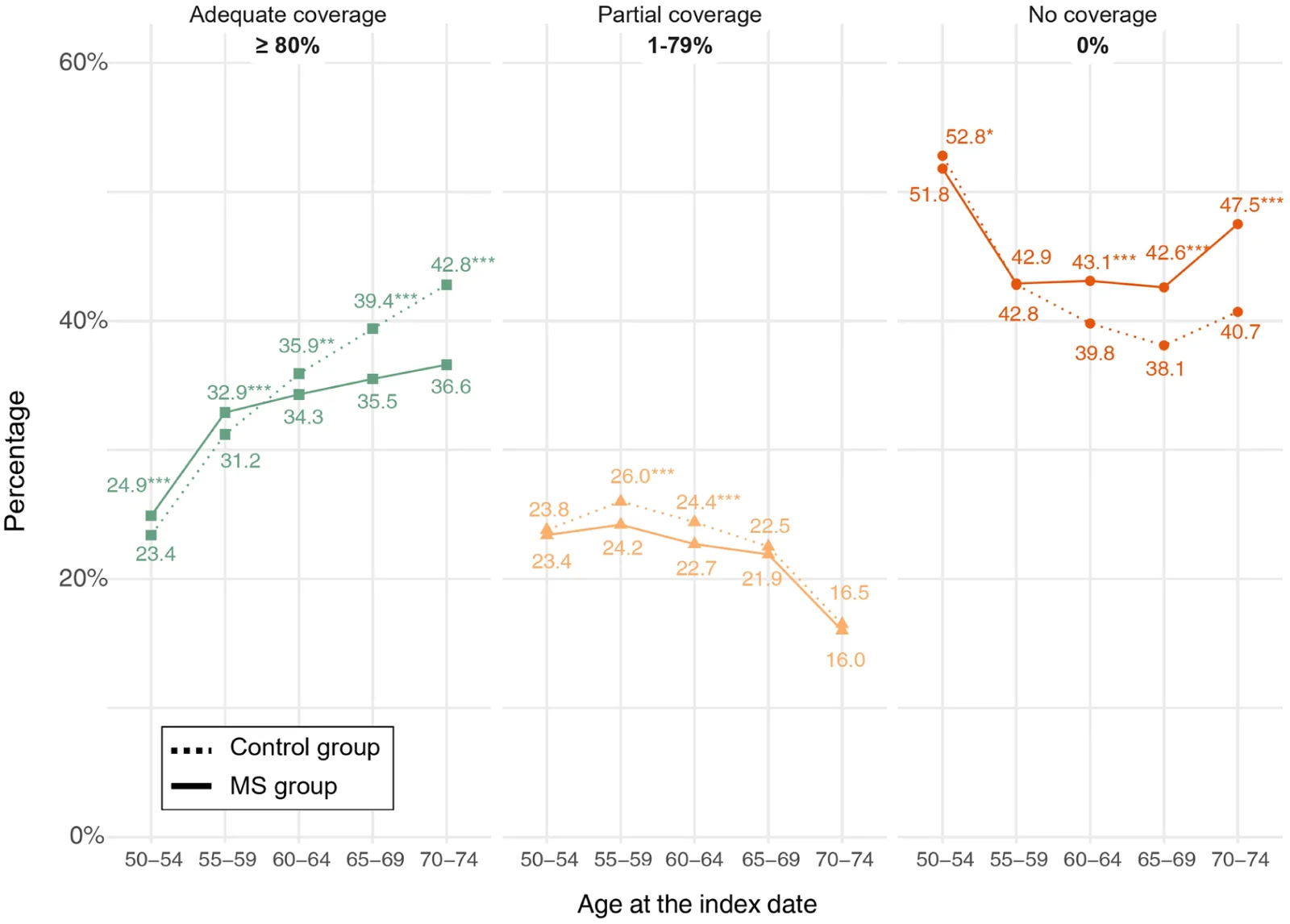
Colorectal cancer screening coverage rates, individuals with MS versus general population controls according to age at the index date. MS: multiple sclerosis. Two-proportion *z*-tests were used to compare each individual category (MS vs control). Significance levels are indicated as follows: *p* < 0.05 (*), *p* < 0.01 (**), *p* < 0.001 (***).

**Figure 3.**
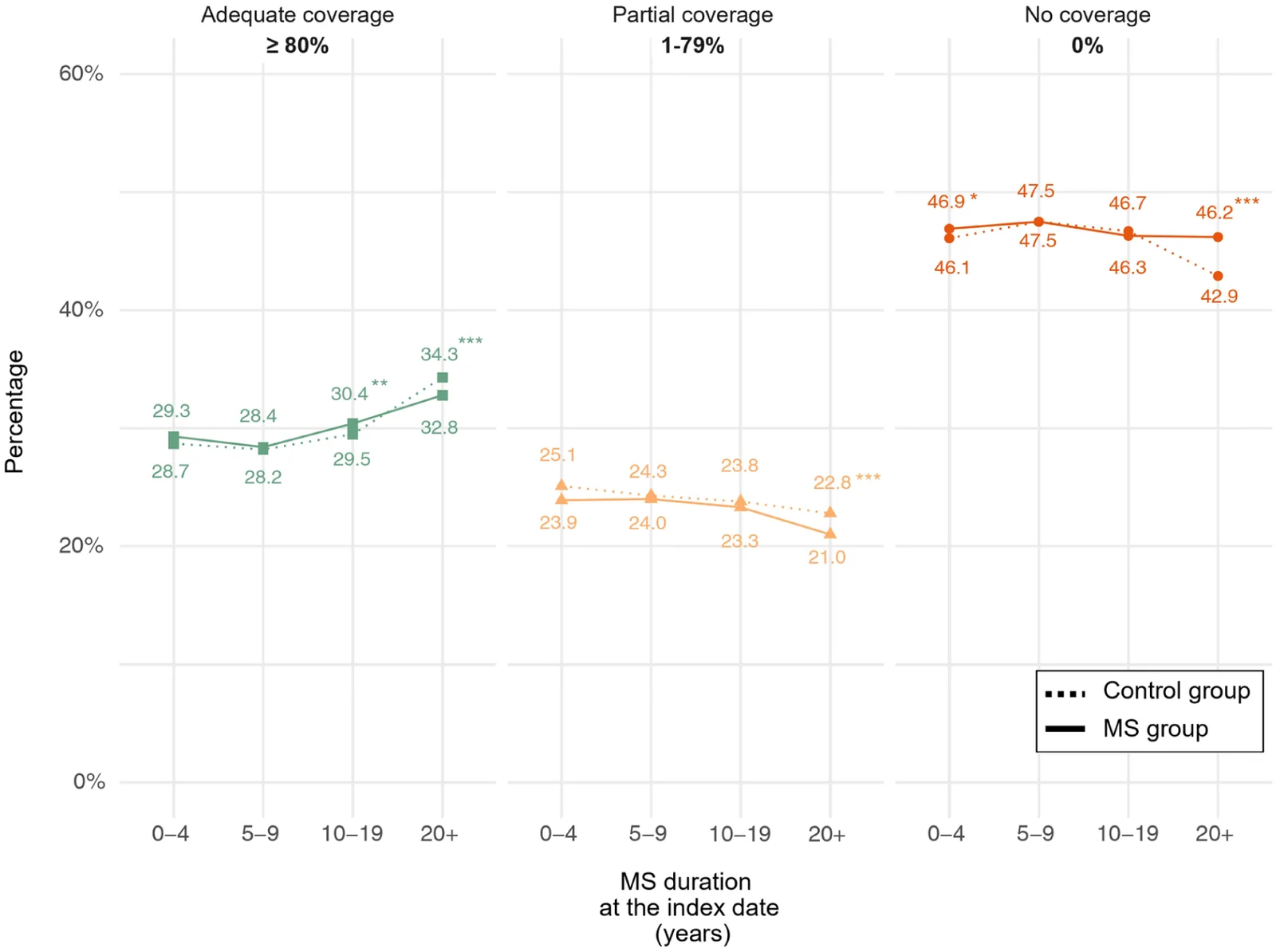
Age- and sex-standardized colorectal cancer screening coverage rates, individuals with MS versus general population controls according to MS duration at the index date in years. MS: multiple sclerosis. Two-proportion *z*-tests were used to compare each individual category (MS vs control). Significance levels are indicated as follows: *p* < 0.05 (*), *p* < 0.01 (**), *p* < 0.001 (***).

Analysis of CCS coverage by age (Figure 2) showed that in the 50–59 years groups, the proportion of individuals with adequate coverage (⩾80%) was slightly higher in pwMS than controls (50–54 years: +1.5%, *p* < 0.001; 55–59 years: +1.7%, *p* < 0.001). Conversely, in the 60–74 years groups, coverage was lower in pwMS. The gap widened as age increased: from a 1.6-point decrease in the 60–64 years group (*p* = 0.001) to a 6.2-point decrease in the 70–74 years group (*p* < 0.0001). Results were similar in both sexes (Figure S1). CCS coverage rates by MS duration showed minimal difference (<1 point) until 19 years of disease (Figure 3). The strongest difference was observed in the proportion of those with no coverage in the ⩾20 years MS duration group (+3.3% for pwMS; *p* < 0.0001).

#### Factors associated with CCS

Crude and adjusted mixed-effect logistic regression models identified factors associated with CCS coverage rate ⩾80% in pwMS (Table 2). Coverage was positively associated with healthcare utilization, including DMT use (OR = 1.12 [1.08–1.16]), at-home nursing visits (1.18 [1.13–1.23]), pharmacy visits (1.81 [1.55–2.11]), and frequent GP visits (3–4 visits/year: 1.21 [1.14–1.27], ⩾5 visits/year: 1.47 [1.40–1.54], ref. 1–2).

**Table 2.** Factors associated with colorectal cancer screening in pwMS, mixed-effects logistic regression model estimates (*n* = 62,373).

	OR [95% CI]			
	Crude	*p*	Adjusted	*p*
Sex		<0.0001		<0.0001
Woman	Ref.		Ref.	
Man	0.83 [0.80-0.86]		0.90 [0.86-0.93]	
Age, years		<0.0001		<0.0001
50-54	Ref.		Ref.	
55-59	1.48 [1.41-1.55]		1.48 [1.41-1.55]	
60-64	1.58 [1.50-1.66]		1.58 [1.50-1.66]	
65-74	1.72 [1.64-1.80]		1.71 [1.63-1.80]	
MS duration, years		ns		ns
0-4	Ref.		Ref.	
5-9	0.97 [0.91-1.03]		0.999 [0.94-1.07]	
10-16	1.02 [0.96-1.07]		1.004 [0.95-1.06]	
20+	1.06 [0.998-1.12]		0.98 [0.92-1.03]	
FDep (index of social deprivation)		<0.0001		<0.0001
First quintile (least deprived)	Ref.		Ref.	
Second quintile	1.02 [0.97-1.08]		0.94 [0.88-0.99]	
Third quintile	1.03 [0.97-1.08]		0.93 [0.87-0.98]	
Fourth quintile	1.00 [0.95-1.05]		0.90 [0.84-0.95]	
Fifth quintile (most deprived)	0.90 [0.86-0.95]		0.81 [0.76-0.86]	
				
CMU status	0.63 [0.59-0.68]	<0.0001	0.68 [0.63-0.73]	<0.0001
Charlson comorbidity index (excluding cancer and hemiplegia)		0.005		ns
0	Ref.		Ref.	
1	1.11 [1.04-1.18]		1.01 [0.95-1.07]	
2	1.09 [0.96-1.24]		1.01 [0.88-1.15]	
3+	1.06 [0.85-1.32]		0.96 [0.76-1.20]	
Hemiplegia	0.73 [0.68-0.78]	<0.0001	0.63 [0.58-0.69]	<0.0001
**Healthcare use in the 12** **months before the index date**				
Number of visits to a general practitioner		<0.0001		<0.0001
0	0.61 [0.57-0.66]		0.71 [0.66-0.77]	
1-2	Ref.		Ref.	
3-4	1.29 [1.22-1.36]		1.21 [1.14-1.27]	
5+	1.57 [1.50-1.65]		1.47 [1.40-1.54]	
Use of at-home nursing services	1.43 [1.38-1.49]	<0.0001	1.18 [1.13-1.23]	<0.0001
Physiotherapist visit	1.24 [1.20-1.28]	<0.0001	1.04 [0.999-1.07]	0.06
Pharmacy visit	3.27 [2.84-3.78]	<0.0001	1.81 [1.55-2.11]	<0.0001
Use of DMT	1.04 [1.01-1.08]	<0.0001	1.12 [1.08-1.16]	<0.0001
MS-related hospitalization, overnight	0.74 [0.66-0.83]	<0.0001	0.76 [0.68-0.85]	<0.0001
Non-MS-related hospitalization, overnight	1.05 [0.998-1.11]	0.06	0.97 [0.92-1.03]	ns

MS: multiple sclerosis; CMU: universal medical coverage; FDep: social deprivation index; OR: odds ratio; CI: confidence interval; ns: not significant (*p* > 0.1).

Random effect on region of residence.

The adjusted model took into account ambulatory hospitalizations.

MS duration, comorbidities, physiotherapy, and MS-unrelated hospitalizations (⩾1 overnight stays) showed no association.

Conversely, social deprivation and disease severity indicators were negatively associated, including CMU status (0.68 [0.63–0.73]), living in the most deprived areas (fifth vs first quintile, 0.81 [0.76–0.86]), hemiplegia (0.63 [0.58–0.69]), and overnight MS-related hospitalizations (0.76 [0.68–0.85]). Male sex was negatively associated (0.90 [0.86–0.93]). Findings were similar when using CCS thresholds of ⩾50% or >0%, except that comorbidity became negatively associated at both thresholds (Supplemental Table S5).

### Qualitative results

Interviews were carried out with 20 pwMS (*n* = 14 women; mean age = 59.6 years, range = 5–72, MS duration range = 1–50, Supplemental Table S6). Ten required the use of a walking aid or wheelchair, and six had limited mobility. Seventeen participants had undergone CCS at least once (FIT or colonoscopy) and 11 reported adherence to the national screening recommendation. Awareness of organized screening campaigns was limited: only 10 were aware of CCS awareness campaigns, and one person knew specifically about the “Colorectal Cancer Awareness Month” campaign known as “Blue March.” The perceived barriers and facilitators are summarized in Table 3.

**Table 3.** Themes related to barriers and facilitators to colorectal cancer screening.

Verbatims		Colorectal screening status
Barriers		
Limited knowledge on colorectal cancer and its screening procedure	“None. Well, I know it exists, that’s all.” (W, 64)	Yes
	“I know it affects the colon and that if it’s not caught in time, it can be dramatic, like any cancer, anyway.” (W, 53)	Yes
	“Not really. I don’t know much about it. Well, I know that if you bleed when you go to the bathroom, there’s a problem. But that’s all.” (W, 59)	Yes
	“I don’t know how to describe it, but I imagine that a diet that is too rich, too fatty, and not varied enough, lacking in fiber or vegetables, could promote the onset of colorectal cancer. Otherwise, no, I don’t know any more than that, since I don’t have it.” (M, 59)	Yes
	“I don’t know if it’s like the mammography, every two years.” (W, 54)	Yes
Limited knowledge on colorectal cancer screening programs and campaigns	“Well, I don’t know anything about Blue March. Well now, I didn’t even know that it existed!” (W, 58)	Yes
	“Sometimes, maybe there’s some information, but, off the top of my head, I can’t really identify them right now.” (M, 56)	Yes
	“Not at all. We talk a lot about breast cancer [. . .]. It hasn’t been in the media for very long either. But for other types of cancer, no, not at all.” (W, 53)	Yes
	“Well, I remember . . . Don’t ask me what, don’t ask me how, but I remember seeing something.” (M, 61)	No
	“At the doctor’s office, at the pharmacy, and at the university hospital. But otherwise, no, I don’t see any, there aren’t any . . . In everyday life, no, I don’t see any, no, not at all, no specific posters.” (M, 59)	Yes
Limited exchanges with friends and family and healthcare providers	“My relatives, my parents, they are over 80, so they don’t do it anymore. But my brother does it. There’s my sister-in-law too. As for my other friends, we don’t talk about it.” (W, 54)	Yes
	“To be honest, I don’t think I’ve talked about it with my [family and friends]. I know one or two people who have done it, but I’ve never really talked about it . . .” (W, 58)	No
	“I think my sister, who is three years older than me, has [undergone screening]. As for my parents, I couldn’t say. I have no idea.” (M, 55)	Yes
	“I don’t think many of my relatives or friends have done it.” (W, 72)	No
	“Actually, I don’t know. It’s not something we talk about much, really. [. . .] My brother, I think [underwent screening], but I don’t know. It’s not something we necessarily talk about.” (M, 56)	Yes
	“Even my general practitioner, when we’re chatting, if I don’t come in with the intention of leaving with a screening kit, no, he won’t [bring it up].” (M, 59)	Yes
	“No, pharmacists, never. Nor would a neurologist, he sticks to his [field]. MS only, nothing else.” (W, 59)	Yes
	“No, I definitely received the prevention leaflet. And then I said to [my doctor], ‘Here, I received this,’ and she gave me the kit. As far as I remember, that’s how it happened.” (W, 54)	Yes
	“It was the practical implementation of the test that put me off, [. . .]. It’s probably good for me, but practically speaking, that’s what put me off, if you like.” (M, 70)	No
	“The idea of what you have to do to take the test doesn’t appeal to me.” (W, 58)	No
Motor and digestive issues related to MS	“Yes, because [. . .] in practical terms, you know, there’s a little piece of paper you have to fill out. And I have a bit of trouble moving around and things like that.” (W, 58)	Yes
	“Yes, because, let me explain, I only have one hand that works. I’m not sure how to get it . . . I got the thing, and I don’t see how it works. So, there you go, it’s not clear to me, so I’m wondering how I’m going to do it.” (M, 61)	No
	“It’s not great. [. . .] Honestly, the collection process isn’t great. Because it’s a small, thin piece of paper. And on top of that, I have trouble standing on the toilet. So, I haven’t done it yet, I’m going to have to do it eventually. It’s not easy.” (W, 59)	Yes
	“And since I often have loose stools, I don’t think it’s worth it.” (W, 59)	No
	“Since MS forces me to spend a lot of time sitting down, I am a little bit constipated.” (M, 70)	No
Feeling of over-medicalization related to MS	“In the same week, I see the psychologist, the psychomotor therapist, the physical therapist, and the nurse. So, it’s true that it’s a bit too much.” (W, 54)	Yes
	“I used to be more serious about it. Less so since I got MS, because I admit I’m a little fed up with . . . Well, I’m a little fed up. Doctors, you know. I’ve been through a lot.” (W, 59)	No
	“I’m going back to more regular appointments, that’s all. Now that I know that if I have [an appointment at the hospital], it’s only every six months, and that’s good [. . .] And now I’m going to start telling myself, ‘OK, now get back into a serious routine for breast and colorectal cancer.’ I don’t do it, but I should.” (W, 59)	No
**Facilitators**		
Prevention due to the presence of an already existing chronic condition (MS)	“I wouldn’t want to have another thing. So, prevention is essential.” (W, 59)	Yes
	“So, knowing that with the treatment I’m on, I’m still at much greater risk than anyone else of getting cancer, we mustn’t bury our heads in the sand. So, it’s true that I’m very, very diligent about this.” (W, 53)	Yes
	“And then, basically, it’s a safety measure, I would say. I mean, I already have MS, so the last thing I need is to get another disease. So, let’s do everything we can, to make sure everything is okay.” (M, 59)	Yes
	“Then I thought to myself, I already have MS. If the rest could wait a little while, that would be good.” (M, 56)	Yes
Trust in the healthcare system and in health prevention	“What prompted me to get screened? On the one hand, perhaps the fear of having it. And then the incentive letter from the primary health insurance fund [. . .] So, I might as well comply with their request. I would feel reassured.” (M, 59)	Yes
	“But still, I want to take preventive measures. I realize that it’s something that’s very important for oneself and for society in general.” (M, 55)	Yes
	“Because it’s a requirement, I mean, they ask me to. I am a responsible citizen, that’s all.” (W, 64)	Yes
	[Interviewer: Why do you get tested?] "I received them [invitation letters] in the mailbox!” (W, 55)	Yes
Adapting the test to overcome symptom-related limitations	First of all, I don’t use it [the stool collection paper]. I use a plastic container because otherwise it’s not possible at all.” (W, 58)	Yes
	“So, for the shower, we put a stool there, but for the toilet, I don’t see what we could do . . . I can get up, but for collecting stool samples for colorectal cancer, I’ll have to find another way, a potty or something. Because I don’t want to fall in the toilet.” (W, 59)	Yes
	“So, my wife is going to explain to me in detail how to do it. Because frankly, I don’t see how I’m going to collect my stool samples. As for putting them on paper, I don’t know . . .” (M, 61)	Yes

M: man; MS: multiple sclerosis; W: woman.

Each quote is associated with the gender and the age of the participant.

#### Barriers

##### Limited knowledge of CRC and organized screening

Knowledge of CRC and screening remained vague and limited. Many felt they had insufficient knowledge on CRC “Nothing. I know it exists, that’s all” and also on prevention campaigns “I don’t remember it at all. I didn’t pay attention.” This was associated with a general lack of discussion about the topic in public and private circles.

##### Limited discussion with healthcare providers and family

CCS was rarely initiated by clinicians. Participants often needed to bring up the topic: “Even my GP [. . .] if I don’t go in with the intention of leaving with a screening kit, he’s not going to . . .” Other healthcare professionals (e.g. neurologists or gynecologists) were not identified as resources, namely due to “pretty time-restricted” consultations. Similarly, none of the participants reported having discussed it with their pharmacist. Participants also reported limited discussion with friends and family outside immediate relatives: “It’s a subject that we don’t talk about much. [. . .] My brother does it.” This contributed to low perceived concern about colorectal cancer.

##### CCS perceived as an uncomfortable and taboo subject

Screening was considered an uncomfortable and “taboo” subject. FIT practical aspects discouraged participation: “The idea of what you have to do to take the test doesn’t appeal to me.” Conversely, BCS was described as “normal,” highlighting social acceptability differences.

##### MS-related motor and digestive issues

Participants described difficulties with hand function and motor skills that complicate the FIT procedure. “He will not be able to do it himself. Because his motor skills in his hands [. . .] are no longer what they used to be” [carer of MS participant]. Digestive issues, which concerned half of participants and included “constipated” and stools that are “too liquid,” influenced the perceived test feasibility.

##### Feeling of over-medicalization

The burden of managing MS contributed to preventive care fatigue, including screening. FIT, even when performed at home, was sometimes viewed as an additional medical obligation as they were “fed up” with medical appointments. Some women reported prioritizing BCS over CCS, indicating a perceived hierarchy of preventive procedures.

#### Facilitators

Participants mentioned wanting to avoid having to manage two chronic illnesses at the same time: “I already have MS, the last thing I need is to catch another disease.” This was part of a general feeling of confidence in the healthcare system. Receipt of a CCS invitation letter from the National Health Insurance also prompted participation: “I received them in my mailbox!.” Some participants interpreted invitations as civic responsibilities of “a responsible citizen,” while others emphasized general preventive health: “I want to take preventive action.” Overall, facilitators were linked to trust in the healthcare system and prevention awareness, rather than CRC-specific concerns. Anticipating worsening mobility limitations, participants noted that the option of receiving home-assistance from a qualified care professional would be valuable.

### Integration

The quantitative and qualitative components showed multiple convergences: trust and use of the healthcare system and physical disability emerged, respectively, as facilitator and barrier in both analyses (Table 4). However, some divergences emerged concerning physical disability. In the quantitative analysis, only hemiplegia was statistically associated with CCS and not MS duration (proxy for accumulated disability). Conversely, only motor and digestive issues were mentioned in the interviews. Moreover, the interviewed pwMS reported feelings of overmedicalization (barrier), but higher healthcare use was statistically associated with greater CCS uptake (facilitator). Finally, restricted health awareness (qualitative) and lower socioeconomic status (quantitative) emerged as complementary barriers because low socioeconomic status was associated with lower health literacy and access to care.

**Table 4.** Integration of quantitative and qualitative results.

Theme	Quantitative	Qualitative	Integration
*Trust and use of the healthcare system*	Visits to the GP, use of at-home nursing services, physiotherapy, DMT use, and pharmacy visit were **positively** associated with CCS.	Trust in the healthcare system and in overall health prevention was **perceived as a facilitator.** Receiving formal invitations to screening from the national health insurance was **perceived as a facilitator**	**Convergence** Trust and interaction with the healthcare system are associated with higher CCS.
*Disability and MS-induced symptoms*	Overnight MS hospitalization and hemiplegia were **negatively** associated with CCS. Comorbidities and MS duration did not **show any association.**	Motor and digestive issues were **perceived as barriers.** Walking-related disability was not mentioned.	**Convergence** Disability seems to be associated with lower CCS. **Divergence/Expansion** Both components show heterogeneous results: walking disability was rarely mentioned in the interviews, but hemiplegia was strongly negatively associated.
*Limited knowledge and discussions*	Lower socioeconomic status (CMU, FDep) was **negatively** associated with CCS. Visits to the GP were **positively** associated with CCS.	Limited knowledge of CCS, colorectal cancer, and lack of discussion in the private and medical spheres were among the most frequently mentioned **perceived barriers**.	**Complementarities** Lower socioeconomic status is associated with lower health literacy and access to physicians. This could further limit knowledge and discussion around CCS.
*Feeling of overmedicalization due to MS*	Healthcare use was **positively** associated with CCS. In pwMS, CCS coverage rate **decreased as age increased** compared with matched controls.	Feeling of overmedicalization was **perceived as a barrier.**	**Divergence** Quantitative results showed that individuals with high healthcare consumption had higher CCS, but it was seen as a barrier in the interviews. **Expansions** Healthcare use accumulation over time might negatively affect CCS in pwMS.

CCS: colorectal cancer screening; CMU: “*Couverture médicale universelle*” [individual indicator of low socioeconomic status]; DMT: disease-modifying therapy; FDe: social deprivation index [ecological]; GP: general practitioner; MS: multiple sclerosis; pwMS: person with multiple sclerosis.

## Discussion

This study found suboptimal CCS uptake in both pwMS and matched general population controls (41.2% and 42.4% in the 2022–2024 period, respectively). Nearly half of each group had no CCS coverage during 2022–2024 (46.2% and 44.5%, respectively). Overall, pwMS had a 3% relative decrease in CCS coverage compared with their matched controls. This difference was primarily driven by older age groups: the gap progressively increased after the age of 60, and reached its peak (−6.2 points) in the 70–74 years group. Conversely, CCS coverage did not vary meaningfully by MS duration. These results confirm the low participation in CCS in the general population in France, like in other high-income countries.^25,26^

Quantitative and qualitative analyses of facilitators and barriers to CCS in pwMS identified trust in and use of the healthcare system as the most convergent factor. Physical disability showed partial convergence (walking disability not mentioned in interviews), suggesting that specific functional limitations, rather than overall disease duration, may influence screening participation and may not be accurately measured in quantitative analyses. Limited CCS awareness and lower socioeconomic status were additional barriers. Although the measured constructs differed, altogether these findings suggest that structural and informational barriers may contribute to screening disparities. Feelings of overmedicalization were reported as a barrier in interviews, but higher healthcare use, including DMT-use, was associated with greater CCS coverage. This may reflect greater healthcare engagement and contact, resulting in more preventive care opportunities. Alternatively, it may reflect the cumulative MS care burden: with increasing age, ongoing disease management may reduce the prioritization of preventive screening despite frequent healthcare contacts.

Similarly, a previous study on BCS in MS by our group showed lower screening uptake and an increasing age-related disparity compared with controls.^12^ BCS uptake remained higher than CCS in pwMS and controls, despite the identification of additional barriers (e.g. long disease duration, fatigue, procedure accessibility, and scheduling). BCS was seen as a routine procedure and healthcare providers were identified as playing a major role in its uptake. Conversely, CCS was perceived as taboo and rarely discussed. Health awareness and knowledge were identified as important facilitators for BCS, but as barriers for CCS. Unlike for BCS, the majority of the identified barriers and facilitators for CCS were related more to the screening procedure than to MS and had been previously identified in general population-based systematic reviews.^27–29^ This aligns with our population-based estimates showing low CCS participation in both pwMS and controls.

A key limitation of this study is the lack of precise quantitative disability data. We relied on proxy variables, such as hemiplegia (only partially identified through hospitalizations) and MS duration, which have limited precision. Research using more precise measures of functional impairment (including spasticity, motor, or sensitive disorders) and digestive symptoms is needed to better assess their association with CCS. For colonoscopies, we could not fully distinguish screening from diagnostic procedures, although we minimized this issue by excluding individuals with a history of inflammatory bowel disease or cancer. Nonetheless, some non-differential residual misclassification may persist. These estimates reflect opportunities for colorectal cancer detection and are likely to overestimate true screening adherence while preserving the internal validity of comparative estimates. Strengths include the use of a population-based database that fully captures healthcare procedures from public and private institutions, thus minimizing selection, reporting, and social desirability biases. The qualitative component added context by capturing the participants’ experiences and perceptions, and the mixed-methods design allowed triangulation, enhancing the validity and providing deeper insights into the mechanisms influencing cancer screening and preventive care in MS. The qualitative sample may underrepresent more socially deprived or isolated pwMS, which may have limited the identification of barriers experienced by underserved populations.

To conclude, CCS uptake remains suboptimal in both pwMS and the general population, particularly in older pwMS. Unlike BCS, most of the identified barriers and facilitators to CCS were not MS-specific. They appear to reflect broader preventive care challenges rather than CCS-specific obstacles. These findings suggest that for CCS, general population-level interventions addressing accessibility and engagement could be prioritized and would also benefit pwMS. As pwMS are aging, new challenges are emerging, including mental and physical disability, complex disease management and increasing comorbidities, and more research on disparities in cancer and general preventive care is needed.

## Supplemental Material

sj-docx-1-msj-10.1177_13524585261465197 – Supplemental material for Colorectal cancer screening in individuals with multiple sclerosis: A mixed-methods study Supplemental material, sj-docx-1-msj-10.1177_13524585261465197 for Colorectal cancer screening in individuals with multiple sclerosis: A mixed-methods study by Chloé Pierret, Romane Bouvier, Sandrine Kerbrat and Emmanuelle Leray in Multiple Sclerosis Journal
