# Supplementary material for: Colorectal cancer screening in individuals with multiple sclerosis: A mixed-methods study
Source: Mult Scler. 2026 Aug 7;32(10):1069–82. doi: 10.1177/13524585261465197 (PMC13538906; doi:10.1177/13524585261465197)
Supplement: Supplementary file 1 [file sj-docx-1-msj-10.1177_13524585261465197.docx]

Table S1. Good Reporting of A Mixed Methods Study (GRAMMS) checklist

| Guideline | Section: page |
| --- | --- |
| Describe the justification for using a mixed methods approach to the research question | Introduction: p.1 |
| Describe the design in terms of the purpose, priority and sequence of methods | Methods: p.2 |
| Describe each method in terms of sampling, data collection and analysis | Methods: p.2-4 [quantitative] p.4-5 [qualitative] |
| Describe where integration has occurred, how it has occurred and who has participated in it | Methods: p.5 |
| Describe any limitation of one method associated with the present of the other method | Discussion: p.11 |
| Describe any insights gained from mixing or integrating methods | Discussion: p.10-12 |

O'Cathain A, Murphy E, Nicholl J. The quality of mixed methods studies in health services research. J Health Serv Res Policy. 2008;13: 92-98.

Table S2. SNDS screening procedures codes and coverage duration

| **Code** | **Code type** | **Coverage duration** |
| --- | --- | --- |
| HGFE001, HGFE002, HGQE001, HGQE005, HHFC001, HHFE001, HHFE002, HHFE004, HHFE005, HHFE006, HHNE001, HHNE002, HHNE003, HHNE004, HHQD445, HHQE004, HHSE001, HHSE002, HHSE003, HHSE004, HJQE001, HHQK484, HHQH365, HHGE010, HHLE005, HHFA004, HHFA005, HHFA021, HHFA022, | CCAM medical procedure code^a^ | 5 years |
| HHQE002, HHQE003, HHQE005 | CCAM medical procedure code^a^ | 10 years |
| HHFA028, HHFA029, HHFA030, HHFA031, HJFC023, HJFA012, HJFC003, HJFC001 | CCAM medical procedure code^a^ | 99 years [lifetime] |
| 1629 | NABM laboratory code^b^ | 2 years (FIT) |
| 9311, 9434, 3217 | primary care reimbursement codes | 2 years (FIT) |

^a^From the list of medical procedure codes used in France: <https://www.ameli.fr/accueil-de-la-ccam/index.php>

^b^From the list of laboratory procedure codes used in France : <http://www.codage.ext.cnamts.fr/codif/nabm/index_presentation.php>

Table S3. Charlson comorbidity index construction (Bannay et. al.)

Source:

“The Best Use of the Charlson Comorbidity Index With Electronic Health Care Database to Predict Mortality” Bannay, Aurélie; Chaignot, Christophe; Blotière, Pierre-Olivier; Basson, Mickaël; Weill, Alain; Ricordeau, Philippe; Alla, François, *Medical Care* 54(2):188-194, February 2016.

doi: 10.1097/MLR.0000000000000471

Adapted Charlson comorbidity index equation from Bannay et. Al. (excl. cancer and hemiplegia):

 2*[congestive heart failure] + [peripheral vascular disease] + [cerebrovascular disease] + 2* [dementia] + [chronic pulmonary disorder] + 2* [mild liver disease] + [mild renal disease] + 3* [moderate or severe liver disease] + [HIV or AIDS]

Table S4. Interview grid

I. Introduction

- Study presentation

- Prevention study within the framework of the INSPIRE Chair

- Interview procedures

II. Interviewee presentation

- Can you introduce yourself?
- Date of disease/diagnosis
- Symptoms experienced/level of disability
- Family situation (presence of a caregiver, child, spouse)
- Geographical situation (rural/urban, distance from healthcare and preventative services)
- Socio-professional situation

III. Care pathway

- How was your disease diagnosed?
- Which healthcare professionals do you primarily interact with?
- Frequency and nature of visits to the GP and neurologist
- Relations with the pharmacist
- Do you mainly talk about MS and its consequences with them, or do you also discuss other topics?
- Prevention (blood tests, smoking, diet, physical activity, vitamin D)
- Medical care (MRI, rehabilitation...)

IV. Knowledge, access and motivation for the CCS program

- Have you ever participated in CCS?
- If yes

When? How often, if more than once?

*If you only took the test once or stopped, why?*

- Too complicated, anxiety-inducing, false positive, colonoscopy

What do you know about this cancer?

Why did you take the test? What were your motivations?

- Encouraged by healthcare professionals (pharmacist, GP)
- Screening campaigns (posters, Blue March, media)
- Invitation letter

Has this screening been discussed with your GP/neurologist?

Did a healthcare professional recommend the test?

- Was it you or the healthcare professional who approached it?
- Reasons for consultation (appointment made specifically or not)
- Prioritization of CCS in relation to other prevention measures and MS (by the healthcare professional)
- Physician's knowledge and communication on this subject
- *If not, would you have liked that a doctor discussed it with you?*

Where did you get the kit? What did you think of it?

Did you encounter any difficulties in accessing or completing the test?

- Aid/Alternatives
- Adaptation tips
- Test performed by a healthcare professional or a caregiver

Were you afraid of a second diagnosis? Why?

What was the result of the test? If positive, did you have a colonoscopy? What was your treatment plan?

*For women: Do you get screened for other cancers (especially breast cancer)? At the same frequency? Why?*

If you were discussing this topic with other people living with MS, what difficulties might they encounter in getting screened? What facilitators could you identify?

Would you like to add anything?

- Otherwise

Why aren't you getting tested? What are the obstacles?

- Level of confidence
- Colonoscopy to be performed if test positive => no desire to do additional tests?
- Accessibility
- Fear of a second diagnosis
- One step in particular (from receiving the test to sending it to the laboratory)

*If the person wished to be tested*

What barriers have you faced?

- MS itself (e.g., autonomy, balance and dexterity problems, lack of time, other priorities)
- Physical barriers (logistics, distance, unsuitable equipment)
- Human and technical skills of healthcare professionals
- Refusal of help by some people
- Health system organization

Were you encouraged to get tested?

- Family & friends
- Healthcare professional
- Follow-ups

What do you know about colorectal cancer?

Are you familiar with the procedures and different steps involved in getting tested? (e.g., where to pick up the kit, results)

Do you still feel concerned by this screening program?

- National Health Insurance CCS letter of invitation
- Close relative who has been tested/affected by cancer
- Not targeted by the campaigns/given your health problems
- Knowledge of the risk level

Have you ever seen any CRC screening campaigns?

- Media coverage/other cancers (esp. Breast cancer awareness)
- Posters in health spaces
- Blue March

What would need to change/be done to allow you to get tested?

- If your doctor/pharmacist had mentioned it, would you have been convinced to get tested? Why?
- Talking about it with people living with cancer/MS
- Another organization type

If you were to discuss this topic with other people living with MS, what difficulties might they encounter in getting screened? What facilitators could you identify?

What role does prevention play in your healthcare pathway?

Have you participated in other cancer screening programs? Why?

Would you like to add anything?

Figure S2. Colorectal cancer screening coverage rates, MS vs control group according to age and sex

Table S5. Factors associated with colorectal cancer screening treshold of 50% and 0% among individuals with MS, adjusted mixed-effects logistic regression model estimates (n= 62,373)

|  | **OR [95% CI]** | | | |
| --- | --- | --- | --- | --- |
|  | **CCS ≥ 50%** | **p** | **CCS > 0%** | **p** |
| Sex |  |  |  |  |
| Woman | Ref. |  | Ref. |  |
| Man | 0.93 [0.90-0.96] | <.0001 | 0.94 [0.91-0.98] | 0.001 |
|  |  |  |  |  |
| Age, years |  | <.0001 |  | <.0001 |
| 50-54 | Ref. |  | Ref. |  |
| 55-59 | 1.41 [1.35-1.47] |  | 1.46 [1.39-1.52] |  |
| 60-64 | 1.44 [1.37-1.51] |  | 1.48 [1.41-1.55] |  |
| 65-74 | 1.47 [1.40-1.54] |  | 1.42 [1.35-1.48] |  |
|  |  |  |  |  |
| MS duration, years |  | ns |  | ns |
| 0-4 | Ref. |  | Ref. |  |
| 5-9 | 1.01 [0.95-1.07] |  | 1.00 [0.94-1.06] |  |
| 10-16 | 1.00 [0.95-1.06] |  | 1.00 [0.95-1.05] |  |
| ≥20 | 0.96 [0.91-1.02] |  | 0.93 [0.88-0.98] |  |
|  |  |  |  |  |
| FDep, index of social deprivation |  | <.0001 |  | <.0001 |
| 1^st^ quintile (least deprived) | Ref. |  | Ref. |  |
| 2^nd^ quintile | 0.93 [0.88-0.98] |  | 0.90 [0.86-0.96] |  |
| 3^rd^ quintile | 0.90 [0.85-0.95] |  | 0.88 [0.83-0.93] |  |
| 4^th^ quintile | 0.85 [0.81-0.90] |  | 0.83 [0.78-0.88] |  |
| 5^th^ quintile (most deprived) | 0.76 [0.72-0.81] |  | 0.74 [0.70-0.79] |  |
|  |  |  |  |  |
| CMU/C2S status | 0.67 [0.62-0.72] | <.0001 | 0.66 [0.62-0.70] | <.0001 |
|  |  |  |  |  |
| Charlson score (excluding cancer and hemiplegia) | | 0.01 |  | 0.006 |
| 0 | Ref. |  | Ref. |  |
| 1 | 0.94 [0.89-0.997] |  | 0.91 [0.86-0.97] |  |
| 2 | 0.90 [0.79-1.02] |  | 0.92 [0.81-1.04] |  |
| ≥3 | 0.79 [0.64-0.98] |  | 0.85 [0.69-1.05] |  |
|  |  |  |  |  |
| Hemiplegia | 0.64 [0.60-0.69] | <.0001 | 0.65 [0.60-0.70] | <.0001 |
| **Healthcare use in the 12 months before the index date** | |  |  |  |
| Number of visits to a general practitioner | | <.0001 |  | <.0001 |
| 0 | 0.72 [0.70-0.77] |  | 0.70 [0.66-0.75] |  |
| 1-2 | Ref. |  | Ref. |  |
| 3-4 | 1.18 [1.13-1.24] |  | 1.18 [1.13-1.24] |  |
| ≥5 | 1.38 [1.32-1.45] |  | 1.38 [1.32-1.44] |  |
|  |  |  |  |  |
| Use of at-home nursing services | 1.19 [1.14-1.23] | <.0001 | 1.21 [1.16-1.25] | <.0001 |
| Physiotherapist visit | 1.02 [0.99-1.06] | ns | 1.02 [0.99-1.06] | ns |
| Pharmacy visit | 1.65 [1.46-1.87] | <.0001 | 1.52 [1.36-1.71] | <.0001 |
| Use of DMT | 1.16 [1.12-1.20] | <.0001 | 1.22 [1.17-1.26] | <.0001 |
| MS hospitalization, overnight | 0.82 [0.74-0.91] | 0.0002 | 0.77 [0.70-0.86] | <.0001 |
| Non-MS-related hospitalization, overnight | 0.94 [0.89-0.99] | 0.01 | 0.92 [0.87-0.97] | 0.0012 |

Note: random effect on region of residence,

The adjusted model took into account ambulatory hospitalization related to MS.

Abbreviations: MS, multiple sclerosis; CMU, universal medical coverage; FDep, social deprivation index; OR, odds ratio; pwMS, people with MS; CI, confidence interval, ns not significant (p>0.1)

Table S6. Characteristics and use of colorectal screening among MS participants in qualitative interviews.

|  | Population |
| --- | --- |
| **Socio-demographic characteristics** | n=20 |
| Age, years |  |
| Mean [SD] | 59.4 [5.4] |
| Min-max | 54-72 |
|  |  |
| Sex |  |
| Man | 6 |
| Women | 14 |
|  |  |
| MS duration, in years |  |
| Mean [SD] | 19.1 [14.0] |
| Min-max | 1-51 |
| Marital status |  |
| Married/ In a civil partnership | 14 |
| Widow/Widower | 1 |
| Bachelor | 5 |
| Children |  |
| Yes | 13 |
| No | 7 |
| Education |  |
| Without a diploma | 2 |
| Highschool diploma | 2 |
| Bachelor's or technical degree | 7 |
| Master's degree or higher | 9 |
|  |  |
| Family history of colorectal cancer |  |
| Yes | 3 |
| No | 17 |
| Job |  |
| Retirement | 4 |
| Unemployed | 5 |
| Part-time work, disability status, or volunteering | 5 |
| Full-time activity | 6 |
| Mobility |  |
| Using a wheelchair | 4 |
| Use of a walking aid | 6 |
| Limited mobility | 6 |
| Unrestricted mobility | 4 |
|  |  |
| Digestive problems |  |
| Yes | 7 |
| No | 13 |
| **Colorectal cancer screening** | |
| FIT or Colonoscopy |  |
| Yes | 17 |
| No | 3 |
|  |  |
| Regular screening |  |
| Yes | 11 |
| No | 9 |
|  |  |
| Awareness of a colorectal cancer screening campaign |  |
| Yes | 10 |
| No | 5 |
| Don't know | 5 |
|  |  |
| Knowledge of the 'Blue March' campaign |  |
| Yes | 1 |
| No | 19 |

Abbreviations: CCS, colorectal cancer screening; MS, multiple sclerosis; FIT, fecal immunochemical test.
